# Design of Polymer Carriers for Optimized Pheromone Release in Sustainable Insect Control Strategies

**DOI:** 10.1002/advs.202304098

**Published:** 2023-12-25

**Authors:** Christoph Hellmann, Andreas Greiner, Andreas Vilcinskas

**Affiliations:** ^1^ Branch Bioresources Fraunhofer Institute for Molecular Biology and Applied Ecology IME Ohlebergsweg 12 35392 Giessen Germany; ^2^ Macromolecular Chemistry II Bavarian Polymer Institute University of Bayreuth Universitätsstrasse 30 95440 Bayreuth Germany; ^3^ Institute of Insect Biotechnology Justus‐Liebig‐University Giessen Heinrich‐Buff‐Ring 26–32 35392 Giessen Germany

**Keywords:** agriculture, controlled release, formulations, insect pheromones, semiochemicals, sustainable plant protection

## Abstract

Semiochemicals such as pheromones play a major role in communication between insects, influencing their spatial orientation, aggregation, defense, and mating. The rational chemical design of precision pheromone‐releasing materials are increased the efficiency of pheromone‐based plant protection agents. Decades of research is begun to unravel the complex communication structures regulated by semiochemicals, from the neuronal perception of specific chemical substances to the behavioral responses in hundreds of species, including many devastating pest insects. This article summarizes the most effective uses of semiochemicals in agriculture, the behavioral responses of selected target species, and controlled‐release strategies based on formulations such as novel fibrous polymer carriers. This study helps scientists, decision‐makers, farmers, and the public understand the importance of appropriate mating disruption techniques that reduce the need for broad‐spectrum insecticides and limit their impact on non‐target and beneficial insects.

## Graphical abstract

Semiochemicals play a major role in communication between insects and are used in sustainable plant protection. In this article, controlled release strategies of insect pheromones are reviewed to emphasize the large improvement potential for plant protection innovations with less damaging effects on biodiversity and, thus for reducing the need for broad‐spectrum insecticides.

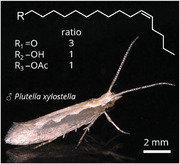

## 1 Introduction

The human population is expected to reach almost 9 billion by 2030, demanding advances in technological areas such as energy, transport, and particularly food production.^[^ ^1^ ^]^ The United Nations (UN) Sustainable Development Goals aim to improve the sustainable utilization of agricultural land and the protection of soil against erosion, ensuring a sufficient supply of nutrients for crops and promoting biodiversity (https://sdgs.un.org/goals). To highlight the urgent need for change, in late 2022 the UN defined 23 additional precise biodiversity targets to be accomplished by 2030.^[^ ^2^ ^]^ However, the extensive use of pesticides, herbicides, and fungicides, which are generally broad‐spectrum synthetic compounds, has caused a dramatic reduction in the number of species and the size of their populations over the last few decades.^[^ ^3^ ^]^ In this review article, we address unresolved challenges facing researchers, decision‐makers, and the public to support Targets 7 (reduce pollution risks), 10 (ensure that biodiversity in agriculture, aquaculture, fisheries, and forestry is managed sustainably), and 21 (ensure the best available data, information, and knowledge are accessible to decision‐makers, practitioners, and the public).^[^ ^2^ ^]^

One route to achieving greater sustainability is the application of biotechnology, such as the use of semiochemicals to reduce the impact of pests while protecting non‐target and beneficial insects like honey bees. In 2021, the global agricultural pheromones market was valued at $US 2.9 billion and is projected to reach $US 6.1 billion by 2026 with existing pheromone application techniques.^[^ ^4^ ^]^ In nature, semiochemicals are emitted by insects to facilitate communication, and many such molecules have been structurally characterized and synthesized for plant protection applications.^[^ ^5^, ^6^ ^]^ The role of these natural semiochemicals (particularly insect pheromones) has been studied for ≈100 years, revealing hundreds of substances representing chemical groups such as mono/di/tri‐unsaturated aliphatic acid esters, ketones, aldehydes, alcohols, epoxides, and hydrocarbons, including complex stereochemical conformations.^[^ ^7^ ^]^ For example, female‐produced attractants have been found in all phylogenetically higher families of moths, and some of these natural semiochemicals have been utilized successfully for pest management, monitoring, and crop protection.^[^ ^8^, ^9^, ^10^, ^11^ ^]^

The major advantages of these typically nontoxic natural substances in crop protection include their activity at low concentrations and their ability to selectively affect target insect behavior, for example by mating disruption, to reduce pest pressure on specific crops. However, one of the greatest challenges in the use of semiochemicals is to ensure the formulation delivers sufficient quantities over an appropriate area and timescale. Many physical and biological factors must be optimized to disrupt insect mating effectively in the field.^[^ ^11^, ^12^ ^]^ Numerous formulation processes are technically suitable for the development of sprayable pheromone dispersions, including organic and inorganic emulsifiers, matrix materials, and embedding agents.^[^ ^13^, ^14^ ^]^ The most promising controlled release behavior has been achieved by embedding semiochemicals in natural or synthetic polymer‐carriers.^[^ ^15^ ^]^

Polymers are already used in agriculture to enhance crop growth under unfavorable conditions by optimizing water and nutrient use, increasing yields, or shortening seasons.^[^ ^16^ ^]^ The success of synthetic polymers reflects the advanced technical and chemical processes for the low‐cost fabrication of large quantities of monomers and polymers with diverse functional properties, including suitable thermal, mechanical, and gas‐permeation characteristics. Most synthetic polymers are used as foils, nets, or covers for agricultural goods, protecting them from weather and animals. Additives can be included during the manufacture of polymers at very low concentrations (<0.5% w/w) to optimize or customize properties related to crystal size and crystal structure, such as viscoelasticity, toughness, mechanical strength, and clearance.^[^ ^17^ ^]^ The functionalization of polymers with semiochemicals may require the synthetic modification of polymer structures to adjust their solubility and phase separation, thus optimizing the release profile while facilitating biodegradation.

**Figure 1:**
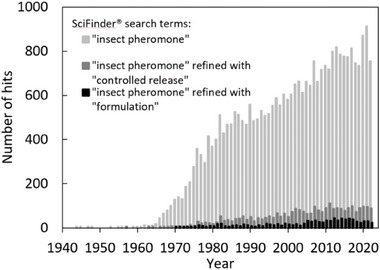
SciFinder hits for the key terms “insect pheromone” alone or with the term “controlled release” or “formulation”. Columns indicate the number of hits per year.

The growing number of scientific publications, proceedings, patents, and book chapters including the term “insect pheromone” over the last eight decades highlights the relevance of this topic, with the number of hits approximately doubling between 1980 and 2020 (**Figure** **1**). Interestingly, the refinement of these hits reveals that the term “controlled release” is present in only 10% of the articles and the term “formulation” in <5%. This proportion has remained constant over time and is mirrored by the conspicuous lack of exploitation, with few applications reaching the market and surviving over time. To explore this phenomenon in more detail, this review summarizes recent achievements in the field of applied agricultural chemistry, especially those involving the chemical design of functional polymers and additives, particularly pheromone–polymer formulations that facilitate the controlled and prolonged release of pheromones. Global challenges related to preserving food security and biodiversity in response to climate change will require dedicated chemists from all fields to find urgent innovative solutions for plant protection.

## 2 Insect Semiochemicals

Semiochemicals play a major role in the intraspecific and interspecific communication of insects for orientation, feeding, defense, and mating. Interspecific substances can be classed as kairomones and allomones, which act as attractors for exploiters (such as parasites) and beneficial symbionts, respectively. Pheromones were originally defined as “Substances which are secreted to the outside by an individual and received by a second individual of the same species, in which they release a specific reaction, for example, a definite behavior or a developmental process”.^[^ ^18^ ^]^ Pheromones induce a wide variety of species‐dependent behaviors that influence sex, aggregation, spacing, alarm, trail following, and maturation, and can therefore be used for the control of insect pests.^[^ ^19^ ^]^

### 2.1 Applications in Agriculture

**Figure 2:**
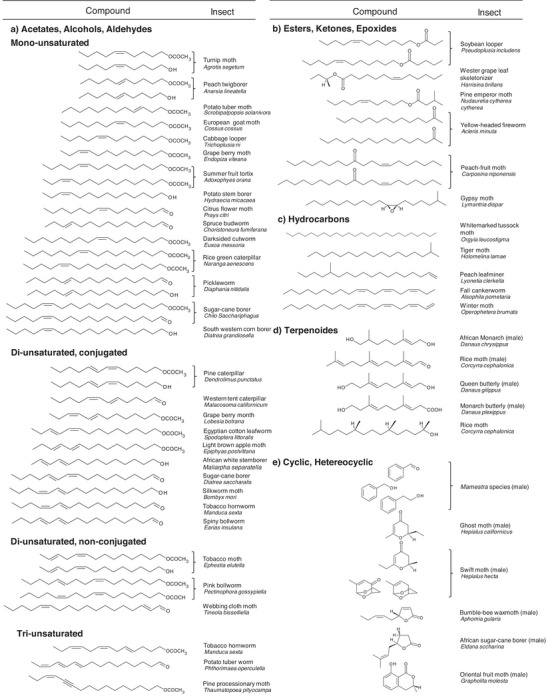
A selection of female and male sex pheromones produced by lepidopteran species indicates the diversity and complexity of the chemical structures. a) Acetates, alcohols, and aldehydes. b) Esters, ketones, and epoxides. c) Hydrocarbons. d) Terpenoids. e) Cyclic and heterocyclic structures. (a–e) Reproduced with permission.^[^ ^9^ ^]^ Copyright 1998, Springer Nature.

Pheromones can induce two behavioral response types: releaser pheromones initiate immediate behavioral responses (such as sex) whereas primer pheromones cause physiological changes (e.g., alarm) that in turn trigger a behavioral response (e.g., protection or attraction).^[^ ^20^ ^]^ Sex pheromones are a subset of releaser pheromones with remarkable long‐range activity, allowing female emperor moths (*Saturnia pavonia*) to attract males up to 11 km away.^[^ ^20^ ^]^ Pheromones have been used in vineyards for mating disruption and thus population control since the late 1930s/early 1940s in Germany.^[^ ^21^, ^22^, ^23^, ^24^ ^]^ In the following decades, pest insect sex pheromones were identified and used to disrupt the mating of various lepidopteran species, revealing numerous insect‐specific chemical structures.^[^ ^24^, ^25^, ^26^ ^]^ Complex chemical enrichment and isolation procedures such as solvent extraction and chromatography were developed to determine these chemical structures and reveal their selective stereochemical information. Some examples of such components, in this case, linear aliphatic and (hetero)cyclic compounds, are presented in **Figure** **2**.^[^ ^9^ ^]^

The fascinating principles of communication and interaction among lepidopteran species are constrained not only by the overall structure of semiochemicals but particularly by the stereoselective configuration, including diastereomeric (R, S) and *cis‐trans* (E, Z) isomeric information.^[^ ^27^ ^]^ For example, pheromone compounds of the spiny bollworm (*Earias insulana*) and the tobacco hornworm (*Manduca sexta*) differ only in the C═C double bond configuration at carbon position 12, namely (E10, E12)‐hexadecadienal and (E10, Z12)‐hexadecadienal, respectively (Figure 2a, di‐unsaturated; conjugated). For comparison, the equivalent alcohol (E10, Z12)‐hexadecadien‐1‐ol is as major sex pheromone in the silkworm moth (*Bombyx mori*), indicating the highly complex relationship between chemical structures and species‐dependent activity.^[^ ^25^, ^28^ ^]^ This relationship at the level of individual compounds becomes orders of magnitude more complex when we consider mixtures of compounds and quantitative variations.^[^ ^29^ ^]^ For example, the pink bollworm (*Pectinophora gossypiella*) sex pheromone is a complex mixture of *cis‐cis* and *cis‐trans* isomers of 7,11‐hexadecadienyl acetate.^[^ ^30^ ^]^

Researchers have focused on lepidopterans because many of the most devastating pest insects belong to this order, including numerous genera of the family Plutellidae, commonly known as diamondback moths. Pheromones in this family include at least three major components: (a) (Z)−11‐hexadecenal, (b) (Z)−11‐hexadecen‐1‐ol and(c) (Z)−11‐hexadecen‐1‐ol acetate. For example, mixtures of (a) and (b) enhanced capture rates compared to the single components in field‐trap assays on stone leek miners (*Acrolepia alliella*), but enrichment trials with (c) showed no further effect.^[^ ^31^ ^]^ Similar findings have been reported for *Plutella xylostella* trap trials, and again the single components (a) and (b) did not capture any males, but mixtures with ratios of 1:4–1:1 significantly increased the capture rates.^[^ ^32^ ^]^ Additionally, capture rates increased with blends of (a) and (b) at ratios of 1:9–9:1, and also with ternary blends of all three components at a ratio of 3:1:1 (also shown in the Graphical abstract).^[^ ^33^ ^]^ Furthermore, the qualitative and quantitative analysis of the pheromone blend emitted by the oriental fruit moth (*Grapholita molesta*) revealed an ≈8:2 ratio of (Z)−8‐dodecenyl acetate (Z8‐12:Ac) and (Z)−8‐dodecen‐l‐ol (Z8‐12:OH). Controversially, release studies from rubber septa revealed an optimum blend ratio between ≈99:1 and ≈9:1, which was needed to match the natural ratio and release rate per female at ≈3 ng h^−1^ Z8‐12:Ac and 0.7 ng h^−1^ Z8‐12:OH.^[^ ^34^ ^]^

Intriguingly, the interplay between different pheromone components and the resulting insect behavioral responses not only reflects the chemical structures, blend ratios, and concentrations but also various seasonal factors such as temperature, humidity, atmospheric pressure, and sunlight/sunset/moonlight.^[^ ^35^ ^]^ For example, interspecific modifications in insect mating behavior were observed in three taxonomically unrelated insects at various natural and experimentally adjusted barometric pressures, especially when the pressure was unstable.^[^ ^36^ ^]^ Accordingly, applying pheromones in fields for mating disruption must take weather conditions and corresponding insect activity into account, and should also aim to control the release rates of the individual components as well as the corresponding blends, but this can be a significant challenge.^[^ ^37^ ^]^ In addition, geographic factors, strain variations, and changing climate conditions play a major role in the success of mating disruption.^[^ ^38^ ^]^ Another improvement strategy involves the use of chemically modified pheromone analogs, known as parapheromones or pheromone structure‐related substances if natural compounds are inefficient under certain field conditions.^[^ ^39^ ^]^ Formulation strategies are also needed to prevent the photochemical oxidation of pheromone molecules in the field.^[^ ^13^, ^14^ ^]^

### 2.2 Toxicology of Pheromones and Commercial Plant Protection Products

The environmental toxicity of natural pheromones produced by insects is considered negligible because the compounds are ubiquitous in the environment and miniscule quantities are sufficient to influence insect behavior (Sections 2.3 and 2.4). These substances can be produced at scale using chemical processes that achieve the necessary stereochemical purity. General chemical safety information is provided by the producers in comprehensive safety data sheets and is also listed in the Globally Harmonized System of Classification and Labelling of Chemicals. However, for plant protection applications, further environmental investigations of technically‐relevant quantities and target insect species need to be taken into account. In the European Union, plant protection products are approved under Regulation (EC) no. 1107/2009, which mandates that newly registered products should be less toxic and induce fewer negative side effects than existing substances and application techniques.

Diverse semiochemical products are applied commercially on at least 10 million ha of land to monitor the presence and/or population density of specific insects and to control certain pest populations by mass trapping, mating disruption, or attract‐and‐kill strategies.^[^ ^6^, ^40^ ^]^ This represents ˂1% of cultivable land, even though intercultural pest insects threaten large cultures, such as moths that attack corn and cabbage fields. Some example products include laminate flakes (Hercon Environmental, USA), twin ampulla dispensers (BASF, Germany), twist‐tie polyethylene dispensers (Biocontroll Shin‐Etsu, Japan), polymer traps/lures (TNO, Pherobank, Netherlands), and sprayable microcapsules (3 M, ISCA, USA, BioPhero, Denmark).^[^ ^41^ ^]^ The first pheromone was registered for pest monitoring in the 1970s. In the 1980s and 1990s, they were used for the first time to cause mating disruption in vineyards, orchards, and tomato, rice, and cotton fields.^[^ ^42^ ^]^ **Table** **1** lists successful examples of mating disruption including pest species, host, materials, and application methods.^[^ ^11^ ^]^

**Table 1:** Successful mating disruption in moths using pheromones and pheromone release systems (adapted from ref. [11]).

Culture/ insect species	Dispenser type/ Formulation	Polymer	Pheromone	First reports	Sources
**Cotton**					
Pink bollworm (Pectinophora gossypiella)	Hollow fibers, twisted rope, microencapsulation	Poly(ethylene), n.a.	(Z,Z)‐ and (Z,E)−7,11‐hexadecadienyl acetates (1:1)	1978, 1981, 1987	[44, 45, 46]
**Orchards**					
Apples / codling moth (Cydia ( = Laspeyresia) pomonella)	Hollow fibers, flakes, tube dispensers	n.a. potentially poly(ethylene)	(E,E)−8,10‐dodecadien‐1‐ol, dodecan‐1‐ol, tetradecan‐1‐ol	1973	[47]
Apples/ light brown apple moth (Epiphyas postvittana)	Twisted rope dispensers	n.a. potentially poly(ethylene)	(E)−11‐tetradecenyl acetate (E,E)−9,11‐tetradecadienyl acetates (20:1)	1986	[48]
Stone fruits / oriental fruit moth (Grapholita molesta)	Tube dispensers	Poly(ethylene)	(Z)−8‐dodecenyl acetate, (E)−8‐dodecenyl acetate, 95:5 (Z) to (E) acetate including 3–10% (Z)‐ 8‐dodecen‐1‐ol	1981	[49]
Black currants/ currant clearwing moth (Synanthedon tipuliformis)	Twisted rope dispensers	n.a. potentially poly(ethylene)	(E,Z)−2, 13‐octadecadienyl acetate (E,Z)−3, 13‐octadecadienyl acetate (100:3)	1987	[50]
**Tomato**					
Tomato pinworm (Keiferia lycopersicella)	Hollow fibers	n.a. potentially poly(ethylene)	(E)−4‐tridecenyl acetate (Z)−4‐tridecenyl acetate 96:4	1978	[51]
**Vineyards**					
European grape moths Eupoecilia ambiguella, Lobesia botrana,	Tube dispensers	Poly(ethylene)	(Z)−9‐dodecenyl acetate, (E,Z)‐ 7,9‐dodecadienyl acetate (1:1.1)	1939, 1985	[21, 23, 24, 52]
North American grape moths Endopiza (= Paralobesia) viteana	Twisted rope dispensers	Poly(ethylene)	(Z)−9‐dodecenyl acetate (Z)‐ 11‐tetradecenyl acetates (9:1)	1984	[53]
**Pheromone release systems**					
(pheromone indicated in light blue, d = diameter)	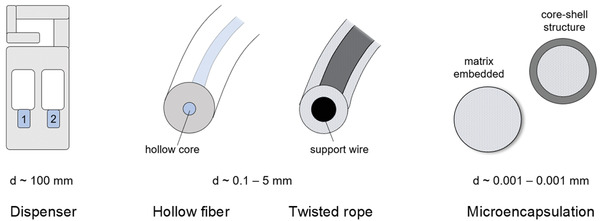				

In typical mating disruption scenarios in vineyards, 500 pheromone dispensers are distributed per ha at the beginning of the flight period of the first generation (BASF Isonet LE), which is one pheromone source per 20 m^2^. These point sources are loaded with 438 mg of pheromone compounds including 205 mg (Z)−9 dodecenyl acetate and 233 mg (E/Z)−7,9 dodecadienyl acetate per dispenser, according to the manufacturer's information. The flight and mating periods of the European grapevine moth (*Lobesia botrana*) and vine moth (*Eupoecilia ambiguella*) last for at least two generations from around April to August depending on weather conditions and the terrain. Under ideal conditions, one dispenser application can disrupt mating for 150–180 days.^[^ ^43^ ^]^ This allows the calculation of a theoretical pheromone release rate of only≈3 mg per day per dispenser, or 1.5 g per ha per day. In comparison, for the mass trapping of oriental fruit moths, an optimal concentration window has been found in field studies (upwind conditions) between ≈7·10^−17^ and ≈2·10^−13^ g cm^−^^3^, equivalent to between ≈7·10^−7^ and ≈2·10^−3^ g ha^−1^ (assuming that each hectare has a vertical dimension of 1 meter: 100 × 100 × 1 m^3^).^[^ ^54^ ^]^ The pheromone concentration required to trap oriental fruit moth males is therefore at least 1000 times lower than that required to disrupt the mating of European grapevine moths and vine moths. Furthermore, mating disruption in the oriental fruit moth was found to be highly effective in numerous countries from ≈1990 onwards.^[^ ^11^ ^]^ These field tests demonstrate successful crop protection strategies using of pheromone approaches that are fully equivalent or superior to protection provided by conventional pesticides.^[^ ^11^ ^]^

An alternative biological insecticide to combat these moths in vineyards is the bacterium *Bacillus thuringiensis* subsp. *kurstaki*, which produces protein toxins known as Bt toxins (Delfin WG, Certis Europe). In this case, ≈600 g per ha of the active substance is applied three times over 21 days, equivalent to ≈85 g per ha per day, which is at least 50‐fold more than the pheromone approaches described above. Furthermore, Bt toxins are selective but not to the same degree as pheromones, so there is a risk to non‐target insects. The general spray application of such insecticides also results in a very high concentration of the active ingredient on day 1 to maintain effective levels until the next application, in this case after ≈7 days.

### 2.3 Insects as Biosensors for Semiochemicals

Insects can perceive volatile semiochemicals at extraordinarily low concentrations (pg per m^3^) with detection thresholds that are orders of magnitude lower than common gas sensors or gas chromatography‐mass spectrometry (GC‐MS).^[^ ^55^, ^56^ ^]^ Intriguingly, the olfactory system of insects may be so selective that certain isomers confer either attractant or repellent effects depending on their spatial configuration.^[^ ^57^ ^]^ For example, the (+) enantiomer of *cis*‐7,8‐epoxy‐2‐methyloctadecane (disparlure) is an attractant for the gypsy moth (*Lymantria dispar*) and the nun moth (*Lymantria monacha*), whereas the (‐) enantiomer causes a selective repellent response in the gypsy moth.^[^ ^58^ ^]^

**Figure 3:**
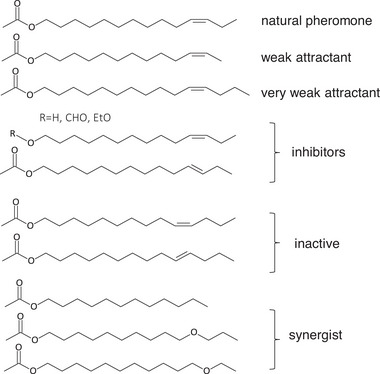
The effect of the natural pheromone (Z)−11‐tetradecenyl acetate (top) and its structural derivatives on red‐banded leafroller (Argyrotaenia velutinana) males. Reproduced with permission.^[^ ^9^, ^59^ ^]^ Copyright 1998, Springer Nature.

The production of such semiochemicals is an energy‐demanding process for the insect, so the lowest possible amounts are produced and released to ensure the conservation of resources. The sensory organs of insects have therefore evolved to become particularly sensitive to specific semiochemicals to avoid false responses to related substances emitted by other species. This ability to differentiate between traces of semiochemicals and odors in natural habitats is remarkable, as indicated by the effects of closely related chemical compounds on male red‐banded leafroller (*Argyrotaenia velutinana*) moths (**Figure** **3**).^[^ ^9^, ^59^ ^]^ The fascinating olfactory capabilities of insects have been exploited to develop bionic models that form the basis of highly sensitive and selective sensor technologies. For example, electroantennography is an ultra‐sensitive sensory technique that can be used to monitor the neuronal response of an insect antenna when traces of volatile semiochemicals are applied.^[^ ^60^ ^]^

**Figure 4:**
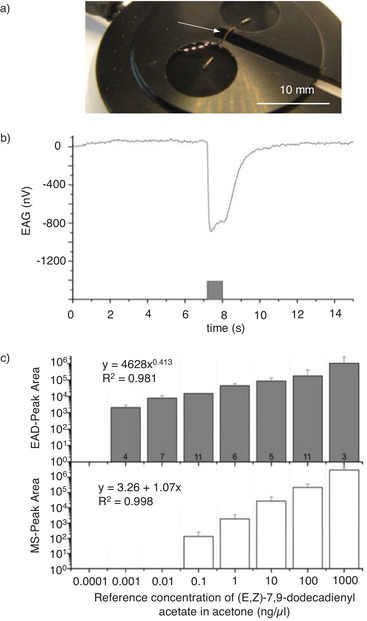
Overview of the electroantennography method. a) Electroantennography setup using a single antenna from a male European grapevine moth and b) the response function triggered by (E,Z)−7,9‐dodecadienyl acetate measured as a drop in potential difference. c) Dose‐response peak area comparison (electroantennography vs mass spectrometry). Peak areas of antennal reaction normalized to 0.1 ng µl^−1^. Reproduced with permission.^[^ ^55^, ^63^ ^]^ Copyrights 2013, Springer Nature.

Such responses are detected by electrodes attached to the whole antenna, the whole insect, or antenna parts.^[^ ^61^ ^]^ Typical electroantennography laboratory setups use a single antenna, for example from a male European grapevine moth, connected to a glass‐isotonic electrolyte electrode (**Figure** **4**a). When exposed to an air puff containing traces of (E,Z)−7,9‐dodecadienyl acetate for 1 s (gray bar), the instantaneous neuronal response of the antenna can be observed and measured by the changing voltage. The peak area integrals of this signal are correlated via a power function over five orders of magnitude with the applied pheromone concentration (Figure 4b,c). Astonishingly, the specific antennal response can be detected at pheromone concentrations as low as 1 pg µl compared to a MS detection limit of 100 pg µl. Accordingly, in this experimental setup, electroantennography was shown to be 100 times more sensitive than the comparative MS technique for the detection of volatile compounds.

The ultra‐sensitive responses of insect antennae to traces of volatile substances indicate the technological potential of novel biosensor systems with orders of magnitude higher sensitivity than current instruments. For example, portable devices for in‐field pheromone concentration measurements could be developed to monitor insect communication during mating activity at a high spatial resolution, allowing farmers to deploy optimized countermeasures.^[^ ^62^, ^63^ ^]^

Such portable devices have been deployed successfully in monocultures such as vineyards, orchards, cotton fields, and eucalyptus groves.^[^ ^64^ ^]^ In contrast to commercial mating disruption via pheromone dispensers in vineyards (Section 2.2), pheromone concentrations of < 100 ng m^−3^ were able to disrupt the mating of European grapevine moths efficiently.^[^ ^65^ ^]^ Additionally, electroantennography combined with GC‐MS identified numerous new compounds for pheromone‐based crop protection in eucalyptus groves.^[^ ^66^ ^]^ The in‐field measurement of pheromone concentrations with high spatiotemporal resolution can improve crop protection by allowing the application of precise pheromone quantities, thus preventing crop damage.

One major challenge affecting in‐field experiments is the differentiation between specific pheromone responses in target insects and general responses to numerous other volatile odors originating from other insects or plants. These observations reveal the complexity of the insect olfactory system, prompting further investigations of characteristic responses to unfamiliar substances. Beyond in‐field pheromone detection, insect‐inspired biosensor devices are being developed to detect traces of hazardous substances such as explosives, drugs, and even volatiles emitted from cancer cells.^[^ ^63^, ^67^ ^]^

### 2.4 Controlling Insect Behavior with Pheromones

Traces of volatile semiochemicals lead to species‐dependent reactions such as the spatial movement of insects approaching or avoiding the source of release. Insect olfactory receptor neurons detect volatile chemicals with various degrees of specificity.^[^ ^68^ ^]^ The volatile odors are usually released by a point source and distributed in the environment by air flows, resulting in Gaussian distribution profiles and odor plumes with broad spatial concentration fluctuations over time.^[^ ^69^, ^70^ ^]^ Many different species detect and respond to these odor plumes for orientation, including moths, mosquitoes, and flies.^[^ ^71^ ^]^

**Figure 5:**
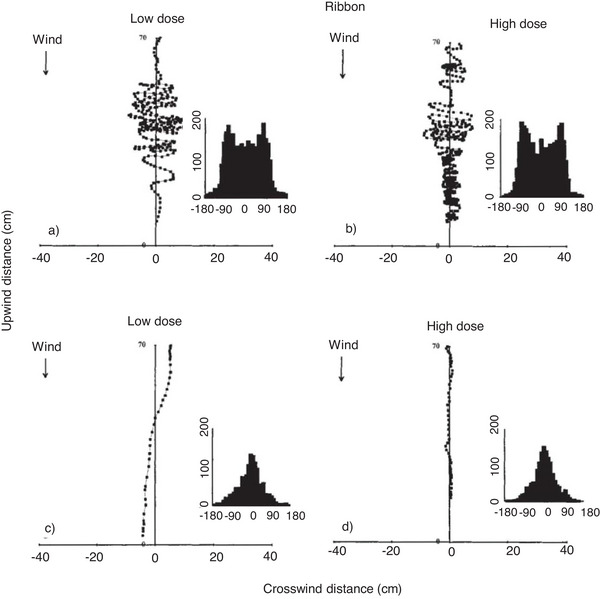
Flight tracks of dried currant moth (Cadra cautella) males in different types of pheromone plume. Ribbon plumes at (a) low and (b) high concentrations produce zigzagging flight tracks whereas turbulent plumes at (c) low and (d) high concentrations produce straight tracks. Accordingly, the frequency distribution histograms of track angles are either bimodal or unimodal. The data points represent the positions of the moth at 0.03‐s intervals. Reproduced with permission.^[^ ^75^ ^]^ Copyright 1994, Springer Nature.

Confirmation that particular compounds mediate communication between insects requires the development and application of suitable bioassays to demonstrate behavioral activity.^[^ ^9^, ^72^ ^]^ These assays use optimized settings adjusted for each species and communication structure of interest, such as alarm, aggregation, or mating.^[^ ^73^ ^]^ Among many available designs for such assays, flight tunnels are the most common. These studies have demonstrated the rapid responses of moths to fluctuations in the concentration of pheromones released from a point source, leading to zigzagging or linear flight tracks along the pheromone plume depending on the concentration pattern following a ribbon‐like, continuous or pulsed, turbulent pheromone release.^[^ ^74^ ^]^ In such crosswind scenarios, the flight tracks appear to be independent of the pheromone concentration because similar tracks were recorded in the presence of 45 or 0.45 ng of the pheromone applied to a filter paper placed in the tunnel (**Figure** **5**).^[^ ^75^ ^]^ Flight behavior can be switched from zigzagging to linear by modifying air flow conditions confirming that navigation and spatial orientation depend on airflow, but other important outdoor parameters such as temperature and pressure also influence odor plumes. In‐field odor plume experiments using negatively charged atmospheric ions combined with electroantennography showed that the antennae of male gypsy moths respond to major spikes within an odor burst (disparlure) at distances of up to 20 m.^[^ ^76^ ^]^ In fields, insects often lose track of the plumes, but numerous experiments have revealed that two main sensory inputs are required: an attractive odor and the wind direction.^[^ ^69^ ^]^

**Figure 6:**
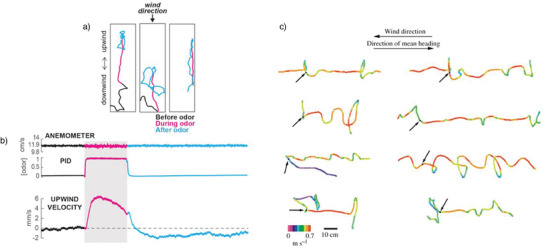
Target‐oriented movement of fruit flies in the presence and absence of an attractive odor. a) Walking tracks and (b) movement velocities of three different flies for ON and OFF responses to an attractive odor before (black), during (magenta), and after (blue) a 10‐s odor pulse. Reproduced with permission.^[^ ^77^ ^]^ Copyrigth 2018, eLife Sciences Publications Ltd, CC BY 4.0 DEED. c) Fruit fly flight pattern and flight velocities during attractive odor release (onset = black arrow). Reproduced with permission.^[^ ^78^ ^]^ Copyrigth 2006, Company of Biologist.

Fruit flies also show target‐oriented movement during flight and walking, as demonstrated in camera‐assisted tracking experiments (**Figure** **6**).^[^ ^77^ ^]^ Onsets and offsets of attractive odor plumes indicating food sources lead to characteristic active response trajectories toward the odor point source (Figure 6a, magenta). When the odor plume is shut off, the flies demonstrate several behaviors as they search for the attractive plume, including movement in small circles at the site where the signal was lost, scanning the arena in larger circles, and walking back in a curved trajectory to the site where the signal was first detected (Figure 6a, blue). When the first plume is detected, the flies show immediate responses in movement velocity (Figure 6b). Similar findings reveal spatial orientation toward attractive odor plumes during flight (Figure 6c). Eight different flight patterns were recorded, representing pronounced and immediate responses (typically within 250 ms) to changes in crosswind direction when an attractive odor plume (fermented bananas, ribbon) was released. Some flies showed straight flight trajectories, whereas others turned in closed loops toward the odor plume of a potential food source with different flight velocities.^[^ ^78^ ^]^ These observations highlight the rapid responses of insects to odor plumes and once again indicate distinctive neuronal capabilities for spatial orientation during the location of food sources and intraspecific communication.

In many moth species, aggregation is triggered by pheromones as an initial long‐range stimulus before close‐range behavior such as mating is induced by other pheromones, whereas in some species the same pheromone controls both behaviors.^[^ ^79^ ^]^ Manipulating the behavior of insects using semiochemicals therefore requires the precise analysis of movement trajectories and changes in responses to single compounds and blends (Sections 2.1 and 2.3). Mating disruption in agriculture requires controlled release to generate numerous plumes of semiochemicals that camouflage natural insect plumes in order to interfere with the natural communication between males and females.

## 3 Controlled Semiochemical Release

### 3.1 Conventional Dispensers and Microencapsulated Formulations

Many different matrices, particularly polymers, have been tested for the controlled release of semiochemicals. For example, single‐component, multicomponent, and multilayered polymer laminate dispensers have been used commercially to release pheromones since the 1980s.^[^ ^8^ ^]^ Such laminates are usually applied as flakes, chips, stripes, or ribbons, and larger quantities can be dropped from aircraft.^[^ ^41^ ^]^ In multilayered materials, the inner layer of active ingredient is protected by two homogeneous polymer film outer layers that control diffusion, dissolution, and gas permeability.

The pheromones released from such matrices typically disrupt male mating behavior using one of three principal mechanisms: 1) they compete with potential mates by causing males to waste time and energy approaching the pheromone sources rather than females; 2) they cause sensory impairment, reducing or even eliminating the ability males to respond to female pheromone tracks; or 3) they create disruptive pheromone “noise” that camouflages the pheromone tracks of calling females. These pheromone formulation approaches are summarized in **Table** **2**, including the application technique, units per ha, and longevity in the field.^[^ ^80^ ^]^ The principles behind the transport characteristics of pheromones released from polymer matrices are primarily described by Hendry's and Fick's laws. For certain active substance and polymer systems, the most important parameters that govern transport are 1) the pheromone concentration in the reservoir and 2) the thickness of the barrier, if the energy of free rotation, free volume, and intermolecular interactions are considered constant. Further parameters that also affect transport properties are related to 3) polymer stiffness, 4) polymer molecular weight, 5) active substance molecular weight, 6) interaction (Flory–Huggins) and solubility (Hansen) parameters, and (7) environmental factors such as temperature, atmospheric pressure and air flow (wind).

**Table 2:** Pheromone formulation strategies for mating disruption in moths using the three principal mechanisms of competition, sensory impairment, and camouflage. Reproduced with permission.^[^ ^80^ ^]^ Copyright 2021, CRC Press, CC BY‐NC‐ND.

Formulation	Density per ha	Application method	Longevity
Atomizer	One or several	Hand‐placed	Season‐long
Sealed polymer tubes	Hundreds	Hand‐placed	Season‐long
Hollow fibers, laminate flakes	∼10000	Aerial	Weeks
Waxy particles	100–10000	Hand‐applied or aerial	Weeks to season‐long
Microcapsules	Millions	Conventional spray	Days to several weeks

**Figure 7:**
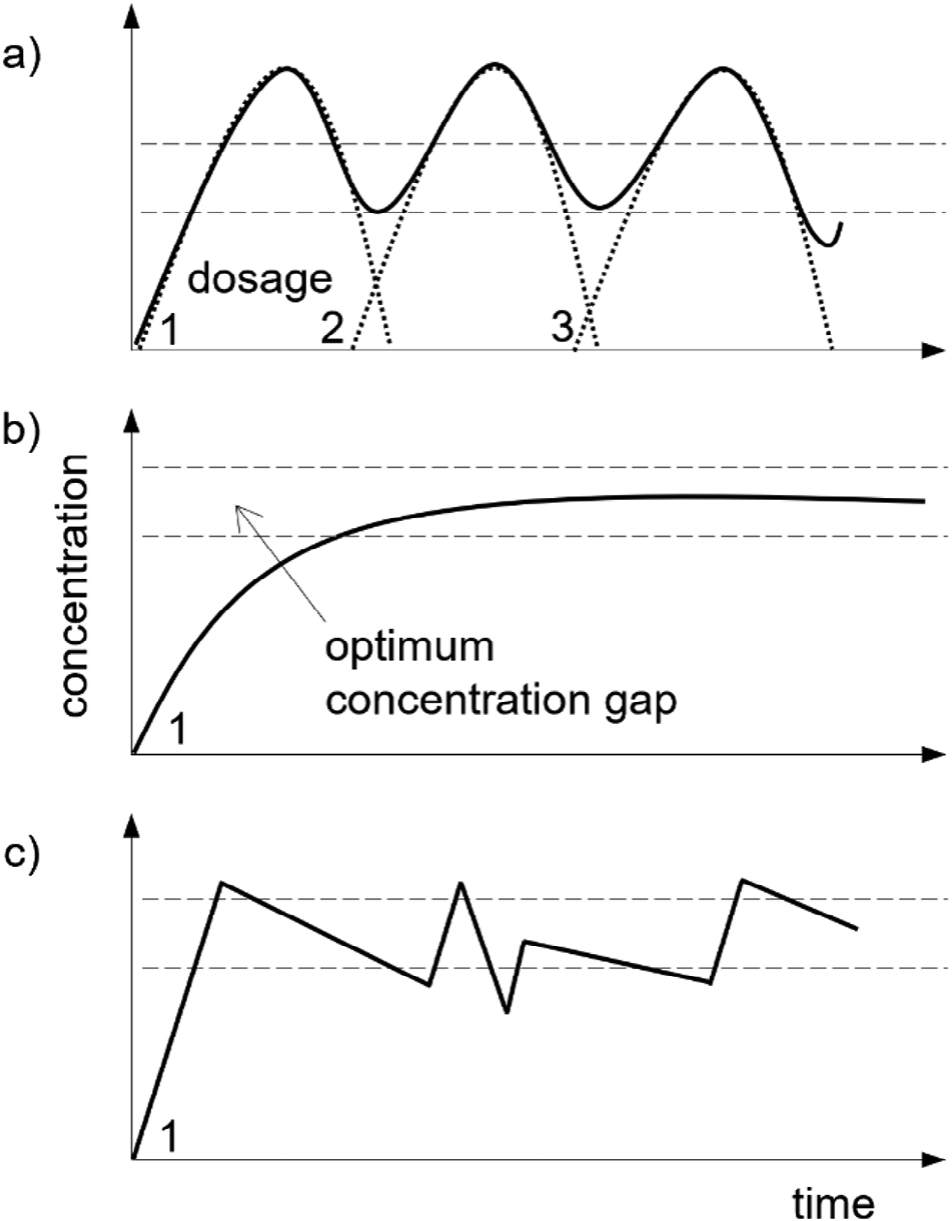
The concentration of pheromones over time is based on different release mechanisms. a) Cumulative concentration based on the application of single doses. b) Controlled release with one dose over time. c) The responsive release mechanism, in which different release characteristics over time can be compensated by triggering the release of more active substances using stimuli such as light, heat, or magnetic pulses.

Most controlled‐release strategies are based on zero‐order kinetics (constant release rate over time). Such equilibrium release kinetics are achieved at the early stage in reservoir–barrier systems because the pheromone concentration in the reservoir is initially high and diffusion is governed by barrier thickness. However, as the pheromone concentration in the reservoir decreases, the system changes toward first‐order release kinetics (decreasing release rate over time). The release characteristics also depend on the choice between multi‐dosage fast‐release systems, diffusion‐controlled release systems, and heterogenic, externally‐induced release systems (**Figure** **7**). The application of active substances with fast‐release profiles has two main disadvantages (Figure 7a). First, a high first dose is typically required to ensure the minimum required concentration is achieved for a suitable length of time, but due to the parabolic shape of the concentration‐time curve, the optimum concentration may be exceeded for short periods . In a pharmaceutical context, this could lead to side effects caused by drug toxicity, and unanticipated effects could likewise be encountered when using pheromones. Second, although the minimum required active concentration is attained quickly, the concentration also falls quickly after peaking, and subsequent (albeit lower) doses are required to ensure the effective concentration is maintained. These issues can be addressed by applying a controlled release strategy, ideally targeting the optimum concentration gap at a constant equilibrium release rate (Figure 7b). An externally‐triggered release principle (Figure 7c) may be suitable if the effect of the active substance is influenced by environmental variables.^[^ ^81^ ^]^ For example, if a pheromone is less active when exposed to direct sunlight, a light‐responsive release mechanism could be used to maintain higher concentrations on sunny days compared to overcast days.

Diffusion‐controlled pheromone release systems based on polymer matrices generally result in first‐order release profiles. To achieve zero‐order kinetics, hollow polymer fibers favor pheromone release from the open fiber ends by capillary forces rather than diffusion through the fiber wall.^[^ ^82^, ^83^ ^]^ The release mechanism involves three steps: 1) evaporation of pheromones at the liquid–vapor surface, 2) diffusion toward the liquid–vapor interface, and 3) pheromone concentration in the inner core. The diffusion step is generally considered rate limiting and can be described using conventional transport equations.^[^ ^84^ ^]^ Hollow fiber dispensers for gossyplure, a 1:1 mixture of (Z,Z)‐ and (E, Z)−7,11‐hexadecadien‐1‐ol, have been shown to achieve linear release profiles under laboratory conditions. Once again, however, some studies have shown that the pheromones were released much more quickly in the field due to the effect of environmental variables such as light, temperature, and wind speed.^[^ ^85^ ^]^ Additionally, conventional spray equipment is not suitable for distributing hollow fiber formulations in the field, resulting in relatively high unit costs for materials, processing and field application.^[^ ^83^ ^]^ Probably for these reasons, hollow fiber approaches are not commercially attractive in particular for large‐area mating disruption and interest has diminished.

**Figure 8:**
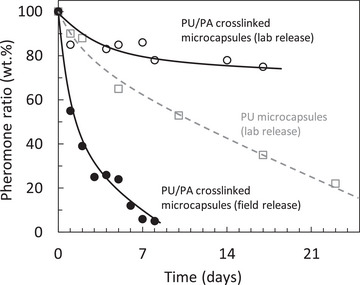
The release characteristics of polyurethane (PU and polyurethane/polyamide (PU/PA crosslinked microparticles. Release profiles of (Z)−9‐tetradecenyl acetate under laboratory and field conditions. Reproduced with permission.^[^ ^88^ ^]^ Copyright 1978, Wiley‐VCH.

Conventional pheromone release systems include laminate dispensers, waxy dispersions, and various polymer systems such as flakes and chips.^[^ ^86^ ^]^ However, the microencapsulation of pheromones is now a major formulation technique that is widely used for mating disruption. Such microparticle systems consist of an inner core containing the highly concentrated pheromone surrounded by an outer shell with tailored diffusion properties.^[^ ^87^ ^]^ For example, microcapsules containing polyurethane (PU) and polyamide (PA) produced by interfacial polymerization have been used since the late 1970s. The release rate of such capsules, ranging from 2 to 10 µm in diameter, is influenced by the wall diffusion parameters, which depend on the degree of chemical crosslinking. The testing of such microcapsules under laboratory and field conditions is shown in **Figure** **8**.^[^ ^88^ ^]^

**Figure 9:**
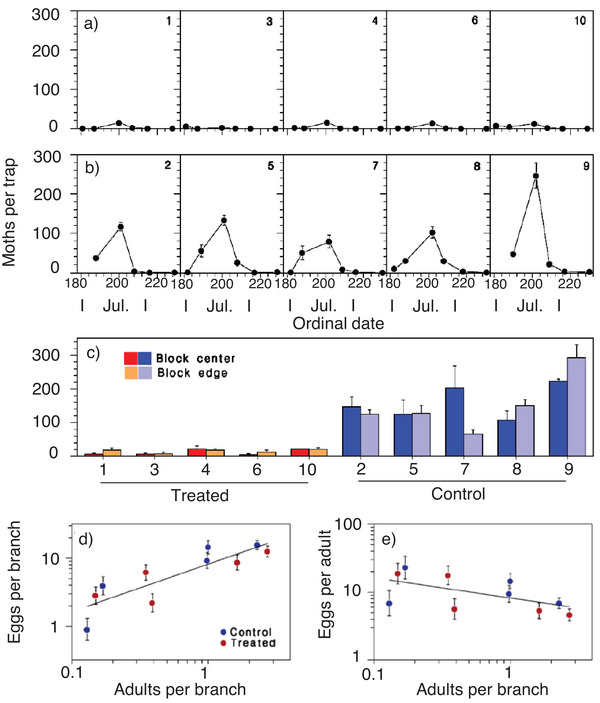
Mating disruption in the eastern spruce budworm (*Choristoneura fumiferana*) using a microencapsulated pheromone formulation on five treated blocks and five control blocks with a 30‐ha block size in Canada, 2021. Mean (± SEM) adult catches in pheromone traps in (a) treated and (b) untreated blocks between June 30 and August 19, including (c) a differentiation between trap catches in the center and at the edges of blocks. d) Egg density and (e) fecundity as functions of pupal case (adult) density. The catch rate reduction in treated blocks was ≈90% compared to the control blocks. However, the relationship between treated and untreated blocks in terms of egg density and fecundity did not reach significance. Reproduced with permission.^[^ ^90^ ^]^ Copyright 2022, MDPI CC BY 4.0.

In contrast to the promising release profiles of cross‐linked polyurethane/polyamide capsules under laboratory conditions with zero wind speed, where the pheromone release rate was slow and reached ≈20% after ≈3 weeks, ≈80% of the pheromone was released within 1 week in the corresponding field trials. The faster release rate under field conditions highlights the strong effects of environmental factors, such as temperature, wind, and sunlight.^[^ ^88^ ^]^ Furthermore, temperature‐dependent increases in pheromone release rates in the laboratory can be ameliorated using paraffin wax and soy bean oil formulations that show zero‐order release kinetics with rates of ≈2 mg day^−1^ over −40 days, but comparative field tests have not been reported.^[^ ^86^ ^]^ Efficient mating disruption was achieved in orchard trials against three insect pests using a conventional sprayable microencapsulated pheromone treatment (3M‐MEC).^[^ ^89^ ^]^ No significant captures of the red‐banded leafroller were recorded over a period of 24 days. The capture rates for the oblique‐banded leafroller (*Choristoneura rosaceana*) increased in the treated plots after ≈14 days although the capture rates in both the treated and untreated (control) plots were comparably low. The capture rates for the oriental fruit moth were at least 50% lower in the treated areas compared to the untreated plot over 24 days. Interestingly, a recent comprehensive study has shown that capture rates alone are not always a sufficient measure to define mating disruption success and efficient plant protection.^[^ ^90^ ^]^ In this recent case, the treatment with a new microencapsulated, sprayable formulation (CONFOUND_SBW_, Andermatt Canada) designed for mating disruption in the eastern spruce budworm (*Choristoneura fumiferana*) showed a very promising 90% reduction in catch rates during field trials. But the analysis of egg: adult ratios after pheromone application revealed no significant reduction in population densities between the control and treatment groups (**Figure** **9**). Even though the population densities before treatment matched required low levels at which mating disruption is considered most effective (< 7 L2 larvae per branch), and the trial was statistically well designed, the authors concluded that 50 g ha^−1^ of active pheromone treatment is not sufficient for successful mating disruption in the eastern spruce budworm.

Another study reported similar ≈90% catch rate reductions using conventional mating disruption flakes but the mating disruption was likewise unsatisfactory and the authors discussed whether > 95% catch rate reductions might be necessary.^[^ ^91^ ^]^ These findings suggest that active pheromone concentrations leading to significantly lower catch rates may still be too low to have a substantial impact on the number of mating events in the field. In the field trial, the application of 50 g active ingredient over 51 days (June 30–August 19) corresponds to a theoretical release rate of ≈1 g ha^−1^ day^−1^, assuming 100% pheromone release with zero‐zero‐order kinetics from the formulation. However, the diffusion‐controlled microencapsulated pheromone formulation is more likely to promote first‐order kinetics, with the release rate declining over time. In comparison, the successful disruption of European grapevine moth and vine moth mating in vineyards requires a ≈50% higher release rate (1.5 g ha^−1^ day^−1^) from a point source (Section 2.2). Again, maintaining the active pheromone concentration profiles in the field over time is a significant challenge for successful mating disruption and requires careful attention during the design of the chemical formulation.

### 3.2 Advanced Pheromone Release from Continuous Polymer Fibers

**Figure 10:**
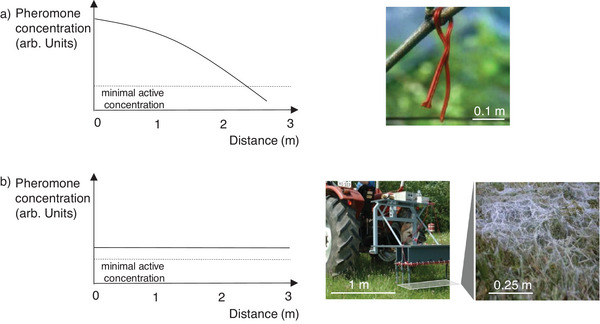
Application of pheromones in vineyards. a) The typical release profile of a common pheromone dispenser‐type point source and (b) optimal release profile of homogenously distributed formulations as an area source with a prototype spinning machine producing fine fibers as potential pheromone carrier systems manufactured directly in the field. Reproduced with permission.^[^ ^92^, ^93^ ^]^ Copyright 2009, Philipps‐Universität Marburg and Copyright 2007, Wiley‐VCH.

Conventional pheromone dispensers are point sources, so the pheromone concentration falls below the minimal amount required for efficient mating disruption at a distance of several meters. This means that dispensers are required approximately every 5 m along the rows in vineyards to maintain a pheromone concentration sufficient to disrupt the mating of European grapevine moths. The pheromone concentration near the dispenser therefore substantially exceeds the required concentration to ensure the minimal concentration is reached midway to the next point source (**Figure** **10**a).^[^ ^92^ ^]^ In contrast, formulated pheromone dispersions that are homogenously sprayed onto the plants represent area sources. Theoretically, the release characteristics of area sources require much lower pheromone doses because sufficient amounts at or slightly above the minimum required level are achieved over the entire area. However, sprayed pheromone formulations have tended to release pheromones too quickly under field conditions (Section 3.1) necessitating further applications every few days. Furthermore, pheromone release is affected by weather, in particular wind and rain, because the particle‐like formulation can be washed off the plants.

Fine fibrous dispenser systems similar in appearance to spider webs have been investigated as an alternative area source to address the drawbacks of sprayed formulations.^[^ ^94^, ^95^ ^]^ Continuous fine fibers can be homogenously distributed in the field using (prototype) machinery, they stick to plants without hindering their growth or access to light, and they are mechanically robust against wind and rain (Figure 10b).^[^ ^93^ ^]^ This approach retains all the advantages of sprayed formulations (e.g., reducing the pheromone dose by a factor of 100–1000 compared to point sources) but is much more resilient. The fibers can be tailored in the same way as other polymer matrices to optimize pheromone uptake and release characteristics, and they degrade naturally to harmless products.

**Figure 11:**
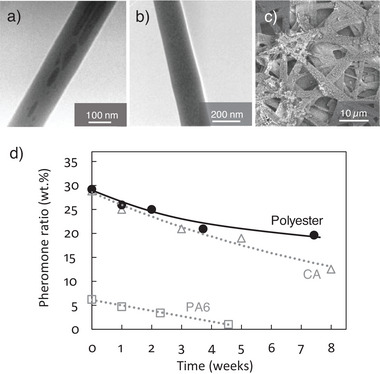
Structure and release characteristics of pheromone‐loaded fibers. TEM images of pheromone‐loaded (a) polyamide 6 (PA6) and (b) cellulose acetate (CA) fibers after staining with ruthenium tetroxide, which increases the contrast of the pheromone. c) Scanning electron microscopy image of pheromone‐loaded polyester fibers. d) The corresponding pheromone release profiles under laboratory conditions (room temperature and constant low airflow) derived from the thermogravimetric analysis of pheromone fibers. Reproduced with permission.^[^ ^92^, ^94^ ^]^ Copyright 2009, Philipps‐Universität Marburg and Copyright 2011, Wiley‐VCH.

In one example, polyamide 6 (PA6), biodegradable cellulose acetate (CA), and a biodegradable polyester (Ecoflex, BASF) were compared as fiber materials to optimize the uptake and release characteristics of (E,Z)−7,9‐dodecadienyl acetate (**Figure** **11**).^[^ ^92^, ^94^ ^]^ Following the selective staining of the pheromone with ruthenium tetroxide, transmission electron microscopy (TEM) revealed phase‐separated pheromone‐rich domains of heterogeneous size and distribution along the PA6 fiber, whereas the CA fibers were characterized by homogenous pheromone‐rich domains 10–20 nm in length. Given the poor solubility of the pheromone in the PA6/formic acid spinning solution, a maximum pheromone load of ≈6% (w/w) was achieved at a yield of ≈30%. Under laboratory conditions (including fume hood air flow), the PA6 system released ≈85% of its incorporated pheromone over 4 weeks. In comparison, the CA/acetone spinning solution achieved a maximum pheromone load of ≈29% (w/w) at a yield of ≈86%. These CA fibers released ≈57% of the pheromone load over 8 weeks in an almost linear fashion. Finally, the polyester/chloroform spinning solution achieved a maximum pheromone load of ≈29% (w/w) at a yield of 88%. These fibers released ≈25% of the pheromone load over almost 8 weeks with near zero‐order kinetics (release rate ≈0.1% (w/w) per day).

Similarly, pheromone‐loaded ethylene–vinyl acetate copolymer fibers produced by solution blow spinning also showed near linear pheromone release profiles over ≈9 weeks at rates of ≈0.05% (w/w) for (Z,Z)−7,11‐hexadecadienal and (Z,Z,E)−7,11,13‐hexadecadienal (pheromones of the oriental fruit moth) and 0.3% (w/w) for (E)−8‐dodecenyl acetate, (Z)−8‐dodecenyl acetate and Z‐8‐dodecenol (pheromones of the citrus leafminer) per day under laboratory conditions.^[^ ^96^ ^]^

In addition, the analysis of residual (Z)−7‐tetradecenal released from CA and polycaprolactone fibers by GC revealed comparable near linear release rates of ≈0.06% ± 0.02% (w/w) per day, albeit at lower pheromone loading efficiencies of ≈25%. The corresponding analysis of the released airborne pheromone by head‐space GC indicated a significantly faster, first‐order release rate with peaks of up to ≈0.9% (w/w) per day (≈0.45 µg pheromone released from a 100 mg fiber mat with a pheromone load of ≈5% (w/w) during 15 min of sample collection).^[^ ^97^ ^]^ Furthermore, electroantennography using oriental fruit moth males exposed to pheromone‐containing nanofibers constructed from different fiber materials showed a near‐linear decrease in antennal activity over a period of 5 weeks under laboratory conditions.^[^ ^98^ ^]^

**Figure 12:**
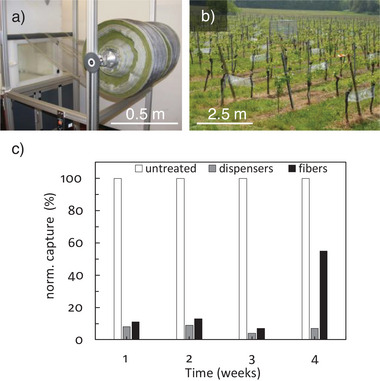
Production and deployment of pheromone‐releasing fibers. a) Technical‐scale production of the pheromone carrier fiber (spun onto a net). b) Placement of trap cage and distribution of fibers in field tests (Freiburg, 2010). c) Catch rate after 1–4 weeks in the field using a living moth as bait. Reproduced with permission.^[^ ^92^ ^]^ Copyright 2009, Philipps‐Universität Marburg.

In field trials using the pheromone/polyester fibers, efficient mating disruption was observed for 3 weeks. These tests involved the use of ≈450 m of hail protection nets coated with fibrous pheromone carriers, with segments ≈1.5 m in length distributed equidistantly over a vineyard area of ≈2000 m^2^ (**Figure** **12**a,b). The distribution of the fibers supported by these nets was not homogeneous and was effectively very similar to the use of point sources, so approximately seven times the amount of pheromone used in conventional dispensers over a period of 100 days was applied to the fibers in this experiment. To investigate the effect of the pheromone‐releasing fibers on the mating of European grapevine moths, a cage with a volume of 2 m^3^ was placed in the center of the test field and loaded with 20–100 male specimens (Figure 12b). A trap baited with a living female was placed at the center of the cage. The effect of pheromone release on mating behavior was monitored by counting the trapped males. The capture rate in blocks of 7 days was compared to a nearby reference experiment in order to account for climate effects. The pheromone‐loaded fibers disrupted mating over a period of ≈3 weeks even without optimization, meaning the distribution was similar to that of multiple point sources (Figure 12c). The performance is likely to improve when the fibers are homogeneously distributed in the field and following the optimization of release characteristics to cover the critical flight period of the target pest. Notably, spray formulations applied as an area source also achieved mating disruption for only ≈3 weeks (Section 3.1). The fibrous pheromone carriers discussed here have not been optimized but currently perform similarly to optimized conventional area sources. This confirms the technical potential of the approach and will guide further improvements in terms of materials, spinning technology, and the in‐field application technique. One such approach is the use of environmentally benign fiber‐spinning technologies such as electrospinning to incorporate and release hydrophobic pheromone compounds.^[^ ^99^ ^]^ The chemical modification of polymers can also improve fiber properties to facilitate the incorporation of hydrophobic compounds such as pheromones and to promote biodegradation.^[^ ^100^ ^]^

Fibrous pheromone carriers are ideal for plant protection when combined with active ingredients such as antifungal compounds, seed coats, or sensor materials for bioactive compounds.^[^ ^101^ ^]^ However, a technology push is required to widen the influence of pheromone‐based plant protection products in agriculture in order to satisfy consumer demands for naturally produced food and other goods. Mating disruption in orchards currently represents the largest market share of agricultural pheromone products, but this remains a specialist area that has yet to be adopted in staples such as corn and other large‐scale crops such as cabbage. The technological advances described in this article will increase the efficiency of mating disruption and other pheromone‐based control methods, boosting the demand for precision pheromone materials and encouraging the deployment of pheromones for the protection of additional crops.

## 4 Conclusion and Perspectives

The inspiring diversity of pheromone‐based plant protection strategies investigated during the past eight decades provides an impressive library of chemical substances that influence the natural communication of insects. The complex interplay between chemical structures and their species‐dependent response profiles has been the topic of numerous previous studies, but only a few pheromone‐based products for plant protection in integrated agriculture are currently market‐ready. Although traces of semiochemicals can be used to direct species‐dependent behavioral responses, mating disruption often fails under field conditions for reasons that remain unclear. Successful pheromone‐based plant protection systems must provide sufficient amounts of active pheromone over time with an appropriate spatial distribution. Currently, ˂1% of cultivable fields are protected using natural pheromones for mass trapping or mating disruption. However, the global agricultural pheromone market is predicted to double from approximately $US 3 to 6 billion between 2021 and 2026, with the strongest market share in orchards. Surprisingly, we found that the most successful pheromone products have changed little since they were developed and launched more than three decades ago. However, we also found a lack of information about new products, including their benefits over previous products, name changes, and reasons for discontinuing certain product lines. We found that pheromone formulations, including sprayable microcapsules and polymer‐carriers, are often let down by their unsuitable release characteristics under field conditions, leading to inefficient crop protection.

To address these issues, key parameters that define important pheromone release properties should be formally described using the interaction (Flory‐Huggins) and solubility (Hansen) parameters of the pheromone and polymer matrix. This would allow the blending of matrix components with high and low pheromone compatibility, leading to phase‐separated domains of pheromone‐rich and pheromone‐poor phases at the microscale or nanoscale. Such formulations could provide useful diffusion barrier properties and reservoir‐like domain structures with tailored release characteristics, resulting in minimal release under humid conditions and at low temperatures (with low insect activity) and faster release under dry conditions and at higher temperatures (with more insect activity). The materials could be prepared from natural and/or synthetic polymers such as polysaccharides, polypeptides and polyesters, as well as biodegradable natural surfactants such as lipids and fatty ingredients (e.g., waxes, aliphatic esters, or alcohols). This would mimic natural solutions, such as the ability of female spiders to release volatile pheromone compounds sequestered in their chitin cuticula and silk webs to attract males.^[^ ^102^ ^]^ Accordingly, we propose that the drawbacks of current formulations can be addressed by designing novel agricultural chemistry products, including new formulation agents, surfactants and additives for release‐optimized pheromone carriers, in particular, to control phase‐separated domains in terms of size and interactions. The challenges must be met urgently in order to 1) reduce the negative effects of broad‐spectrum pesticides, 2) secure food production despite the emergence of pesticide‐resistant insects, and 3) adapt to incipient climate change effects that will significantly affect food security. As a result, additional efforts from academic and industrial researchers are required to develop market‐ready products over the coming decades.

Insect biodiversity is closely related to the socioeconomic wealth of societies, including the switch to sustainable foods and materials. In the next few decades, we must therefore enhance the management of global resources, which includes the development and application of sustainable plant protection systems that protect ecosystems. We conservatively estimate that the value of a strong ecosystem, including the preservation of insect diversity, equates to more than US$ 1 trillion per annum, indicating the need for the entire agrifood sector to seize the opportunity for the adoption of sustainable agricultural practices, including the optimized deployment of insect pheromones to protect crops.

## Conflict of Interest

The authors declare no conflict of interest.
